# Observation of strongly entangled photon pairs from a nanowire quantum dot

**DOI:** 10.1038/ncomms6298

**Published:** 2014-10-31

**Authors:** Marijn A. M. Versteegh, Michael E. Reimer, Klaus D. Jöns, Dan Dalacu, Philip J. Poole, Angelo Gulinatti, Andrea Giudice, Val Zwiller

**Affiliations:** 1 Quantum Transport, Kavli Institute of Nanoscience, Delft University of Technology, Lorentzweg 1, 2628CJ Delft, The Netherlands; 2 National Research Council of Canada, Ottawa, Ontario, Canada K1A 0R6; 3 Politecnico di Milano, Dipartimento di Elettronica Informazione e Bioingegneria, piazza Leonardo da Vinci 32, 20133 Milano, Italy; 4 Micro Photon Devices, Via Stradivari 4, 39100 Bolzano, Italy

## Abstract

A bright photon source that combines high-fidelity entanglement, on-demand generation, high extraction efficiency, directional and coherent emission, as well as position control at the nanoscale is required for implementing ambitious schemes in quantum information processing, such as that of a quantum repeater. Still, all of these properties have not yet been achieved in a single device. Semiconductor quantum dots embedded in nanowire waveguides potentially satisfy all of these requirements; however, although theoretically predicted, entanglement has not yet been demonstrated for a nanowire quantum dot. Here, we demonstrate a bright and coherent source of strongly entangled photon pairs from a position-controlled nanowire quantum dot with a fidelity as high as 0.859±0.006 and concurrence of 0.80±0.02. The two-photon quantum state is modified via the nanowire shape. Our new nanoscale entangled photon source can be integrated at desired positions in a quantum photonic circuit, single-electron devices and light-emitting diodes.

## Abstract (web summary)

Semiconductor quantum dots embedded in nanowires are good candidates for the realization of a nearly ideal entangled photons source. Here, Versteegh *et al.* demonstrate emission of single-photon pairs from a position-controlled nanowire quantum dot without the need for temporal post-selection.

## Graphical abstract

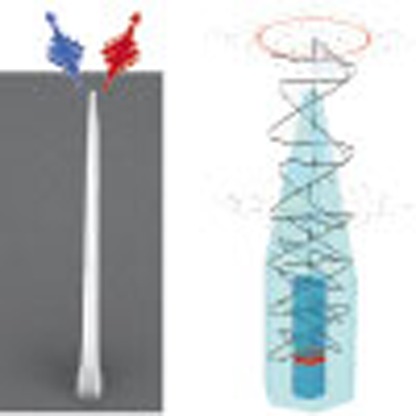

There are demanding requirements for an ‘ideal’ entangled-photon source for implementing ambitious schemes in quantum information processing, such as that of a quantum repeater^1^. The source should meet the following criteria: high brightness combined with high-fidelity entanglement^2^, on-demand generation^3^, high extraction efficiency^4^, directional^5^ and coherent emission^6^, as well as position control at the nanoscale^7^. It is extremely difficult to meet all of these requirements in a single device. Good candidates are semiconductor quantum dots embedded in nanowires.

The high refractive index of a nanowire waveguide around a quantum dot ensures that the emitted light is guided in the desired direction and a tapered end makes the light extraction very efficient^4^. With such a design, efficient single-photon generation has been demonstrated from a single nanowire quantum dot^5^. In addition, the emission mode-profile was shown to be directional and Gaussian^8^,^9^, a key requirement for efficient long-distance quantum communication in well-established telecommunication technology. Nanowires can be controllably positioned in uniform arrays^10^,^11^, with the ability to independently control the dot size and waveguide shell around it^12^. Silicon segments and substrates can be included in the design^13^,^14^,^15^,^16^ and electrical contacts have been demonstrated on single nanowires for single-electron devices^17^, light-emitting diodes^18^, as well as single-photon avalanche photodiodes^19^. A significant advantage of using nanowire waveguides for efficient light extraction over other existing approaches, such as optical microcavities^20^, is the broad frequency bandwidth of operation^21^, which is needed for achieving bright entangled photon-pair generation via the biexciton–exciton radiative cascade. This approach is especially advantageous for quantum dots emitting over a large spectral range and may also be implemented with II–VI quantum dots where the biexciton binding energy is very large (>20 meV)^22^.

A key feature of nanowires with embedded quantum dots grown in the [111]-direction is that the fine-structure splitting is expected to vanish^23^, which should result in excellent entangled photon emission via the biexciton–exciton radiative cascade^24^. Our measurements realize this prediction and demonstrate the generation of strongly entangled photon pairs for the first time from a nanowire quantum dot. Our sources are ready to implement in advanced quantum information processing schemes without the need for any post-growth manipulation^25^ or temporal post-selection^26^. Temporal post-selection can be a major source of photon losses and puts additional requirements on the measurement, thus limiting the scalability of quantum dot-based entangled photon sources. For practical applications it is therefore very useful that we can avoid temporal post-selection. Finally, due to the efficient waveguiding and the tapered end, which we created during the bottom-up growth of the nanowire, we measure a light extraction efficiency of 18±3% for the source. Importantly, due to a recent breakthrough in the nanowire growth^12^, this high efficiency is obtained while potentially meeting all of the criteria of an ideal entangled photon source.

## Results

### Site-controlled quantum dots in tapered nanowire waveguides

**Figure 1:**
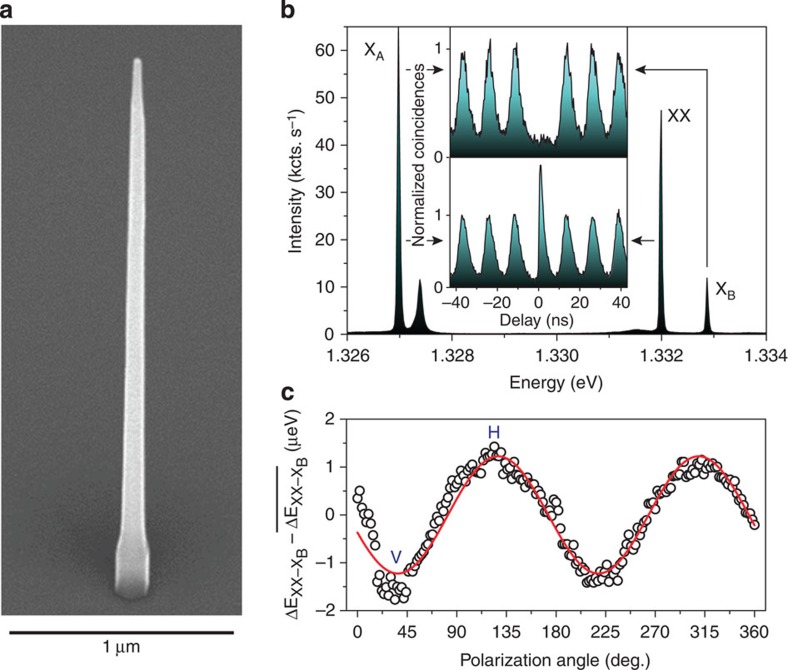
Nanowire quantum dot sample. (**a**) Scanning electron microscopy image of a tapered nanowire waveguide with embedded quantum dot. (**b**) Photoluminescence spectrum of a single InAsP quantum dot embedded in an InP nanowire. The spectrum was taken at the excitation power used for the cross-correlation measurements needed to reconstruct the density matrix (100 nW), which is close to saturation of both XX and X_B_ transitions. Note that the excitonic transition X_A_ saturates the CCD camera. (**c**) Polarization-dependent measurement to determine the excitonic fine-structure splitting. To increase the accuracy of the polarization measurement we plot the relative difference between biexciton XX and exciton X_B_ emission energy. The amplitude of the sine-function fit indicates a fine-structure splitting of 1.2 μeV.

The nanowires were grown by selective-area chemical beam epitaxy, which allows for control of the dot size and position, as well as enabling growth of the waveguide shell around the dot for efficient light extraction (see Methods). This technique has been demonstrated to yield defect-free, pure wurtzite nanowires, which is essential to obtain long single-photon coherence^27^. Figure 1a shows a scanning electron microscopy (SEM) image of a tapered InP nanowire waveguide containing an InAsP segment, 200 nm from the nanowire base, defining the optically active quantum dot that we study.

A spectrum taken under the excitation condition used for the quantum-state tomography measurements is depicted in Fig. 1b. By performing cross-correlation measurements^28^, shown in the inset, and power-dependent measurements (see Supplementary Fig. 1), we identified the biexciton (XX) and exciton (X_A_ and X_B_) transitions. The XX–X_B_ cascade produces entangled photons. In contrast, a weak cascade is observed for XX–X_A_, which does not show entanglement. From these observations, X_A_ could be either a charged exciton or an exciton with a different hole state than X_B_ as is permitted by the wurtzite crystal structure. The transitions XX and X_B_ are resolution limited; single-photon interference measurements show excellent coherence of our entangled photons (see Supplementary Fig. 2 and Supplementary Note 1). Autocorrelation measurements at saturation of the XX and X_B_ transitions show strong antibunching, indicative of nearly perfect single-photon pairs from the XX–X_B_ cascade (see Supplementary Fig. 3).

### Light extraction efficiency

From the single-photon detector counts, 55 kilocounts per second for XX and 15 kilocounts per second for X_B_, under pulsed excitation at 80 MHz, we calculate a collection of 7.9 million XX and 2.0 million X_B_ photons per second into the first objective when taking our 0.7±0.1% setup efficiency at ~900 nm into account. The X_A_ transition shows the highest intensity of 12.1 million photons per second and saturates our spectrometer’s CCD camera with integration times as short as 1 s under the excitation conditions used throughout our study (Fig. 1b). This radiative recombination pathway from XX competes strongly with the X_B_ emission, thus reducing the entangled photon-pair generation efficiency. Taking into account this competing recombination pathway for XX, we calculate a light extraction efficiency of 18±3% for the source. We expect that adding a gold mirror with thin dielectric layer below the nanowire will boost the efficiency nearly twofold^21^. Combining this mirror with further engineering of the nanowire shape promises extraction efficiencies exceeding 90% (ref. 29).

### Low fine-structure-splitting system

Using polarization-dependent measurements, presented in Fig. 1c, we obtain an estimation for the excitonic fine-structure splitting, *S*, by subtracting the XX transition from the X_B_ transition energy^30^. We obtain from the sine-function fit a fine-structure splitting of 1.2 μeV. In the case of nanowires, the small fine-structure splitting is a result of growth on a [111]-oriented substrate and the symmetric hexagonal cross-section of the nanowire core, defining the quantum dot. This small value that we measure for the fine-structure splitting is crucial for the entanglement observation between XX and X_B_ photons without any temporal post-selection and is representative of the sample where, remarkably, a high percentage (>50%) of the measured quantum dots show a fine-structure splitting below 2 μeV (Supplementary Fig. 4). The period of precession of the X_B_ spin can be estimated as *h*/*S*=3.5 ns (ref. 31), where *h* is Planck’s constant. This period of precession is much longer than the X_B_ lifetime of 0.50±0.01 ns as extracted from the XX–X_B_ cross-correlation measurements without polarization selection (Supplementary Fig. 5). Therefore, the X_B_ spin precession has only little influence on the correlations in polarization between the two photons.

### Polarization-entangled photon pairs

**Figure 2:**
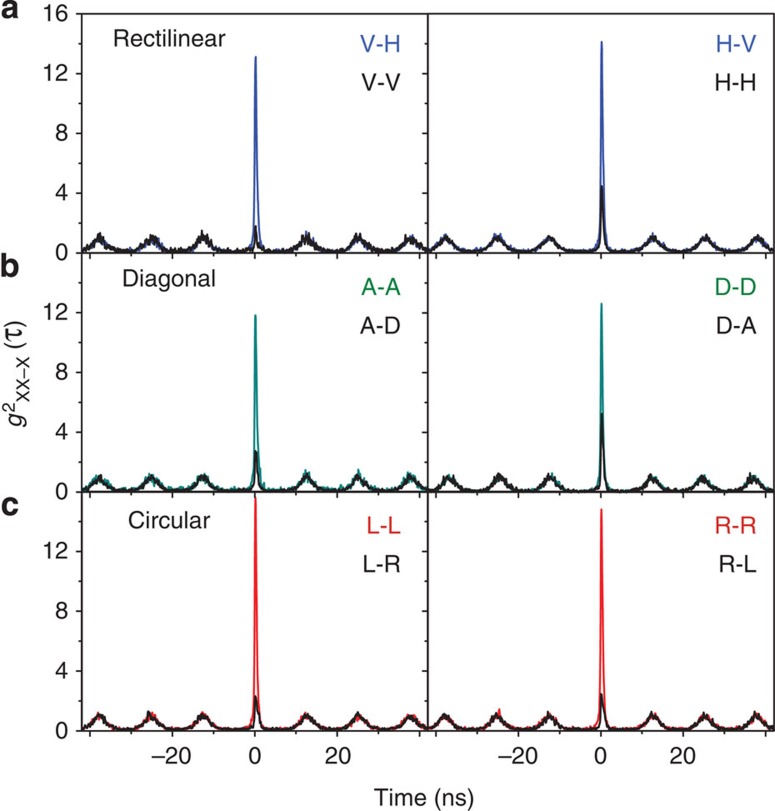
Cross-correlation measurements for the three different bases: (**a**) Rectilinear, (**b**) diagonal and (**c**) circular. The plotted data are normalized to the Poisson level of the side peaks. Start: biexciton; stop: exciton X_B_. The first letter stands for the measured polarization of the biexciton photon, whereas the second letter stands for the measured polarization of the exciton photon.

Twelve cross-correlation measurements in the rectilinear, diagonal and circular polarization bases are shown in Fig. 2, where each histogram is composed of 64 ps time bins. In the correlation measurements, the first letter stands for the measured polarization of the XX photon, whereas the second letter stands for the X_B_ photon. The strong correlations in *HV*, *VH*, *DD*, *AA*, *RR* and *LL*, together with the weak correlations in *HH*, *VV*, *DA*, *AD*, *RL* and *LR*, show that the two photons from the XX–X_B_ cascade are entangled. Here, *H* and *V* are orthogonal linear polarizations (horizontal and vertical), 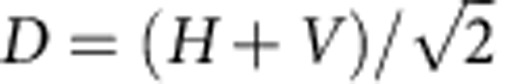 and 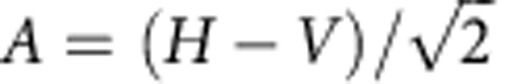 are diagonal and antidiagonal linear polarizations, whereas 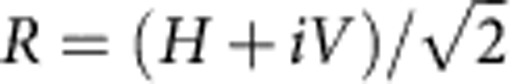 and 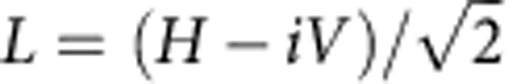 are righthanded and lefthanded circular polarizations.

The quantum state we observe is different from the state that is measured for self-assembled quantum dots^31^,^32^,^33^,^34^,^35^. Typically, one measures for the XX–X cascade bunching (positive correlations) in *HH* and *RL*, and antibunching (negative correlations) in *HV* and *RR*. However, we observe the opposite (Fig. 2a,c). Only in the diagonal basis we see the usual correlations: bunching in *DD* and antibunching in *DA*. These results show that the two-photon quantum state is closer to 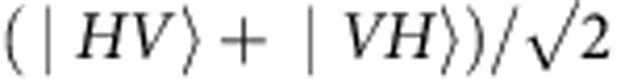 than to the commonly measured state 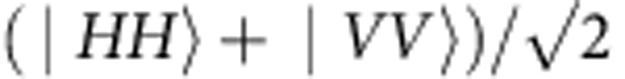 (refs 7, 25, 32, 33, 34).

### Quantum-state tomography

**Figure 3:**
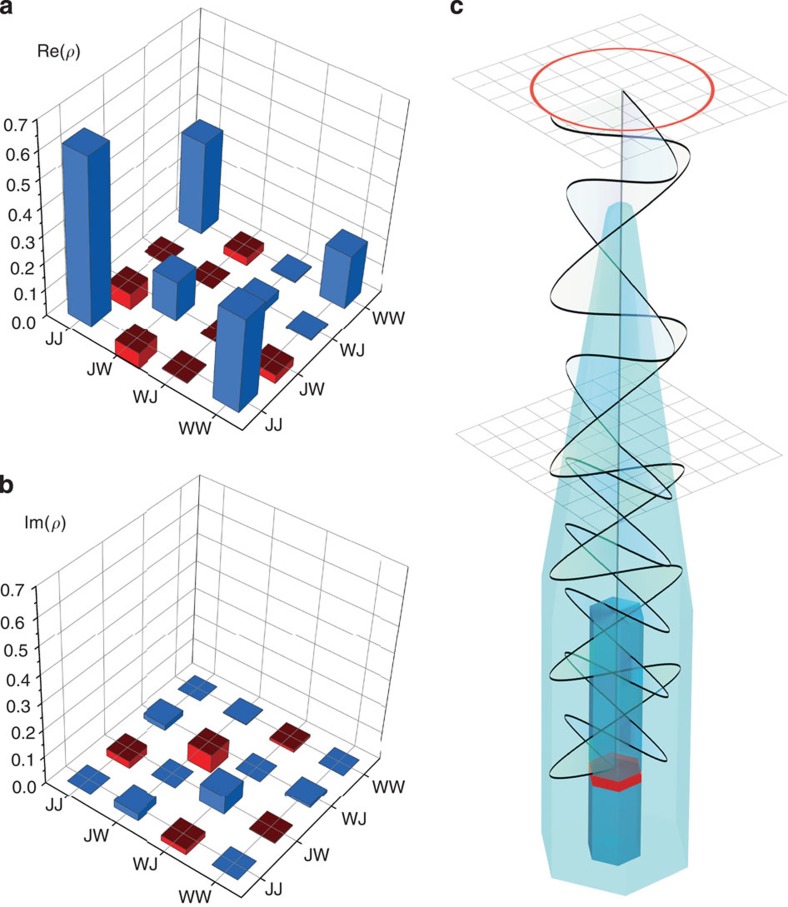
Quantum-state tomography. Real (**a**) and imaginary part (**b**) of the density matrix for the full time window of 6 ns, in the rotated basis. The positive matrix elements are blue, and the negative matrix elements are red. (**c**) Illustration of the effect of birefringence in the nanowire. The orthogonal waves inside the nanowire experience different refractive indices, and therefore their wavelengths inside the waveguide are unequal. As a result, the polarization of the light emission by the quantum dot (red) is modified leading to a different quantum state. The tapered section of the nanowire is more symmetric and is free of birefringence.

We performed quantum-state tomography^36^ to determine more precisely the quantum state of the photons and the degree of entanglement. The raw cross-correlation measurements needed to reconstruct the density matrix are shown in Supplementary Fig. 6. The resulting density matrix is given in Fig. 3a,b. The concurrence is 0.57±0.02. A positive value for the concurrence means that the correlations cannot be explained classically and that the photons are quantum entangled. In this calculation, all correlation counts in the full time window of 6.02 ns are taken into account. The two-photon state has a fidelity of 0.762±0.002 to the maximally entangled state 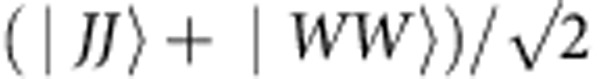, where *J*=*He*^−*iβ*^ cos*α*+*Ve*^−*iβ*^ sin*α* and *W*=−*He*^*iβ*^ sin*α*+*Ve*^*iβ*^ cos*α* are two orthogonal elliptical polarizations. The angles *α* and *β* are specified in Table 1. The classical limit is 0.5, so this result shows a strong degree of entanglement, even without temporal selection.

Selection of a narrower time window yields higher values for the concurrence and the fidelity (Table 1). For example, for a time window of 0.13 ns we calculate a concurrence of 0.80±0.02 and a fidelity of 0.854±0.006. The density matrix for this time window is presented in Supplementary Fig. 7. Temporal selection yields stronger entanglement, because within a narrow time window the effects of spin precession and dephasing processes are smaller^31^. When we do not restrict our analysis to states of the form 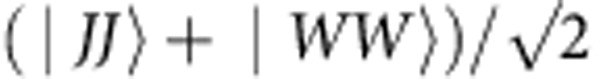, but instead calculate the fidelity to a general maximally entangled two-photon state, we find only slightly higher values (Table 1). The maximally entangled states to which the fidelity is maximal are very close to states of the form 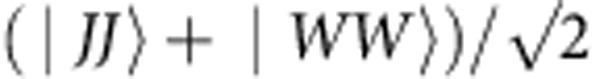.

### Two-photon quantum state modified by birefringence

**Figure 4:**
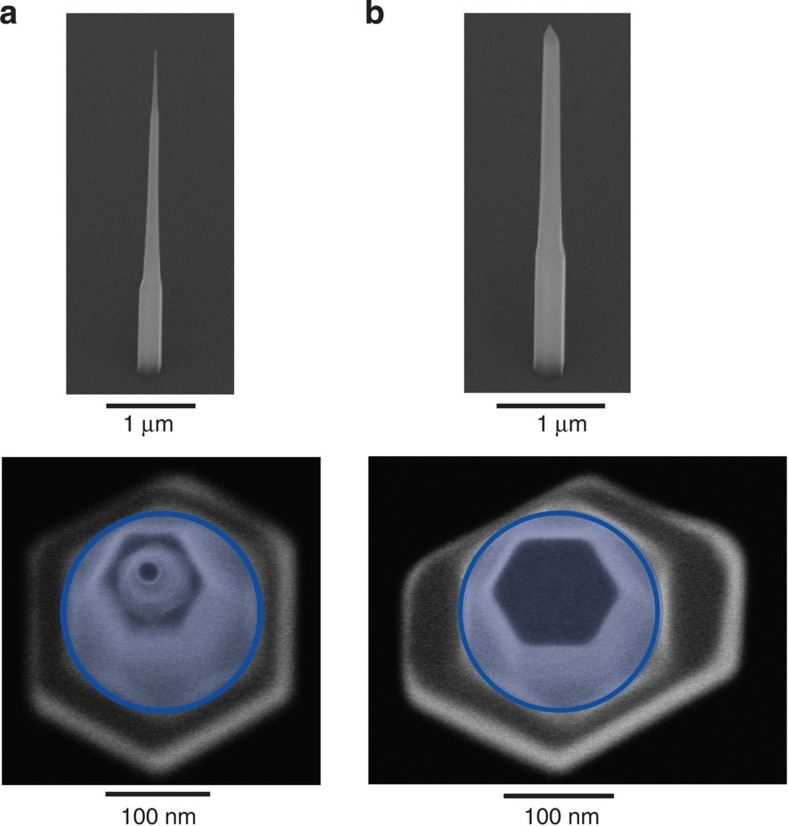
Nanowire birefringence. SEM images of: (**a**) symmetric nanowire waveguide, and (**b**) asymmetric nanowire waveguide. Top panel: side-view SEM images of nanowires with a tilt angle of 45 degrees. Bottom panel: SEM images of the nanowires viewed from the top at a small tilt angle. The blue-shaded circle represents the opening in the SiO2 mask. The example of the nanowire elongation in **b** is an extreme example that leads to geometric birefringence and corresponding rotation of the quantum state.

Why do we measure 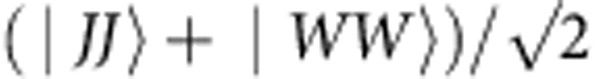 and not the usual two-photon state that is measured for quantum dots, namely 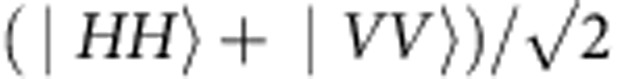
? The most probable reason is that the nanowire has a small anisotropy: it could have a slightly elongated cross-section. An extreme case is shown in the SEM image of Fig. 4b. Such an anisotropy may be formed during the growth of the cladding around the core, and would then be unrelated to the shape of the quantum dot (for details of the growth, see the Methods section). As a comparison, we show a symmetric nanowire waveguide in the SEM image of Fig. 4a. In case of an elongated cross-section the effective refractive indices are different for the polarizations along the short and the long axis of the nanowire. Here, we could imagine that the quantum dot emits photon pairs in the usual entangled quantum state 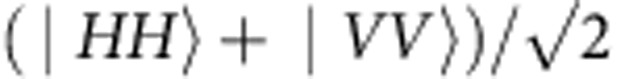. As the emitted photons are guided along the nanowire, the two-photon state is modified by birefringence into 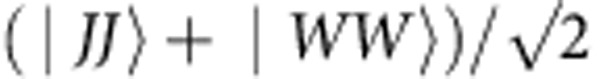, as is illustrated in Fig. 3c. Thus, *HH* and *VV* correlations rotate into predominantly *RR* and *LL* correlations, while *RL* and *LR* turn mostly into *HV* and *VH*, which explains the observations of Fig. 2. For a nanowire waveguide of 6 μm length a difference of effective refractive index of order 0.1 would be enough to explain the magnitude of the observed rotation. Apart from birefringence in the waveguide, the polarization state of the emitted photons could also have been influenced by Γ_7_ and Γ_9_ hole mixing in the wurtzite quantum dot.

## Discussion

In summary, we used a wurtzite nanowire quantum dot to generate single pairs of polarization-entangled photons with a fidelity as high as 0.859±0.006 and a concurrence up to 0.80±0.02. Furthermore, a high degree of entanglement is maintained (fidelity of 0.762±0.002) without any temporal post-selection. This first observation of entangled photon-pair generation from a nanowire quantum dot, which combines the desired properties of an ideal entangled photon source, opens new opportunities in quantum optics, integrated quantum photonic circuits^37^,^38^ and quantum information processing.

To realize an ideal entangled photon source in future work there are several properties of our source to consider. First, quantum dot-entangled photon sources have not yet reached the fidelity or concurrence values of parametric down-conversion sources^2^,^39^. However, with recently available post-growth tuning methods to bring the fine-structure splitting of almost any quantum dot near zero^25^ and two-photon resonant excitation^3^, the fidelity of these quantum dot sources are approaching that of parametric down-conversion sources. Second, the single-photon coherence of the emitted photon pairs is not yet Fourier-transform limited, which is needed for advanced quantum information-processing schemes. Such Fourier-transform-limited photons may be reached by combining two-photon resonant excitation techniques^3^,^40^, cooling of the quantum dot sample to 300 mK^28^ and by accelerating the quantum dot emission via the Purcell effect^20^. Finally, the major advantage of tapered nanowire waveguides over other approaches is the light extraction efficiency, which promises entangled photon-pair extraction efficiencies exceeding 90% due to the broadband frequency of operation^29^. Such efficiencies would surpass the state-of-the-art entangled photon-pair efficiency of 12%^20^, without the stringent requirements needed to engineer both the exciton and biexciton into resonance with a cavity mode by using post-growth manipulation of pre-selected quantum dots.

Note: Similar work is reported by Huber *et al*.^26^

## Methods

### Nanowire quantum dot growth

The InP nanowires containing single InAsP quantum dots were grown using chemical beam epitaxy with trimethylindium and pre-cracked PH_3_ and AsH_3_ sources. The nanowires were grown on a SiO_2_-patterned (111)B InP substrate consisting of circular holes opened up in the oxide mask using electron-beam lithography and a hydrofluoric acid wet-etch. Au was deposited in these holes using a self-aligned lift-off process, which allows the nanowires to be positioned at known locations on the substrate^41^. The thickness of the deposited gold is chosen to give 20-nm to 40-nm diameter particles, depending on the size of the hole opening. The nanowires were grown at 420 °C with a trimethylindium flux equivalent to that used for a planar InP growth rate of 0.1 μm h^−1^ on (001) InP substrates at a temperature of 500 °C. The growth is a two-step process: (i) growth of a nanowire core containing the quantum dot, nominally 200 nm from the nanowire base, and (ii) cladding of the core to realize nanowire diameters for efficient light extraction (around 200 nm). The quantum dot diameters are determined by the size of the nanowire core. In this study, we investigated quantum dot diameters ranging from ~25 to 30 nm.

The nanowire core was grown for 26 min at a PH_3_ flow of 3 s.c.c.m. The dot was incorporated by switching from a PH_3_ to an AsH_3_ overpressure for 3 s after 15 min of growth. This growth time results in a quantum dot height of ~6 nm as determined in our previous studies^12^, using an energy-dispersive X-ray spectroscopy line scan along the nanowire, for a sample with nominally identical growth conditions. We note that our quantum dots are grown during 3 s, resulting in taller quantum dots with longer emission wavelength, as compared with the work of Huber *et al.*^26^ who used a growth time of 2 s. Our growth conditions result in very small fine-structure splittings as shown in Supplementary Fig. 4.

The nanowire cladding was grown by increasing the PH_3_ flow to 9 s.c.c.m. The total growth time was 120 min. To realize the smooth tapering towards the tip, the nanowire was made longer than the diffusion length of indium. Most nanowires, including the quantum dots, have a pure wurtzite crystal structure. The nanowire axis is the wurtzite *c* axis.

### Nanowire waveguide elongation

Radial growth is nominally constrained by the oxide opening, and the nanowire cross-section has a hexagonal symmetry. Optimal coupling of the quantum dot emission to the waveguide mode requires diameters exceeding that of the oxide opening. This is achieved by increasing the cladding growth time, which results in the nanowire overgrowing the oxide opening. No longer constrained by the opening, the hexagonal symmetry may be distorted (see Fig. 4). This asymmetry results in a geometric birefringence and concomitant rotation of the polarization state emitted by the quantum dot.

### Optical measurements

The optical measurements were performed in a standard confocal microscopy setup where the quantum dot sample is cooled to a temperature of T=5 K in a closed-cycle cryostat. The setup consists of two spectrometers both equipped with red-enhanced single-photon avalanche diodes having 75 ps time resolution, dark count rates as low as 80 counts per second and quantum efficiency of 11.5% at 930 nm^42^. A set of waveplates and polarizers placed in front of each spectrometer was used to perform polarization-dependent cross-correlation measurements. One spectrometer is set to the biexciton (XX) transition and the other to the exciton (X_B_) transition. Each correlation measurement was done with 6,000 s of integration to reach over 1,000 correlations in each side peak. For all photoluminescence and correlation measurements, we use a Ti:Sapphire laser emitting 3-ps-long pulses at 750 nm with a repetition rate of 80 MHz to excite the quantum dot.

## Author contributions

M.E.R., M.A.M.V. and V.Z. conceived and designed the experiments. K.D.J., M.E.R. and M.A.M.V. performed the experiments. D.D. and P.J.P. fabricated the sample. M.A.M.V., M.E.R. and K.D.J. analysed the data. A.Gu. and A.Gi. developed the detectors. M.E.R., M.A.M.V., K.D.J. and V.Z. wrote the manuscript with input from the other authors.

## Additional information

**How to cite this article**: Versteegh, M. A. M. *et al.* Observation of strongly entangled photon pairs from a nanowire quantum dot. *Nat. Commun.* 5:5298 doi: 10.1038/ncomms6298 (2014).

## Supplementary Material

Supplementary Information Supplementary Figures 1-7 and Supplementary Note

## Figures and Tables

**Table 1:** Calculated concurrences and the fidelities for five different time windows.

**Time window (% of coincidence counts)**	**Concurrence**	**Fidelity to** 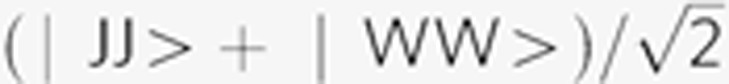	**Fidelity to general maximallyentangled state**
6.02 ns (100)	0.57±0.02	0.762±0.002 (*α*=33°, *β*=46°)	0.765±0.002
1.40 ns (80)	0.64±0.02	0.793±0.002 (*α*=33°, *β*=47°)	0.796±0.002
0.70 ns (59)	0.69±0.02	0.814±0.003 (*α*=34°, *β*=48°)	0.818±0.003
0.38 ns (35)	0.73±0.02	0.839±0.003 (*α*=35°, *β*=49°)	0.842±0.003
0.13 ns (10)	0.80±0.02	0.854±0.006 (*α*=36°, *β*=50°)	0.859±0.006

The fraction of the correlation events taken into account for a certain time window are given in brackets in the first column.

## References

[b1] Briegel H.-J., Dür W., Cirac J. I. & Zoller P. Quantum repeaters: the role of imperfect local operations in quantum communication. Phys. Rev. Lett. 81, 5932–5935 (1998).

[b2] Kwiat P. G., Waks E., White A. G., Appelbaum I. & Eberhard P. H. Ultrabright source of polarization-entangled photons. Phys. Rev. A 60, R773–R776 (1999).

[b3] Müller M., Bounouar S., Jöns K. D., Glässl M. & Michler P. On-demand generation of indistinguishable polarization-entangled photon pairs. Nat. Photon. 8, 224–228 (2014).

[b4] Claudon J. *et al.* A highly efficient single-photon source based on a quantum dot in a photonic nanowire. Nat. Photon. 4, 174–177 (2010).

[b5] Reimer M. E. *et al.* Bright single-photon sources in bottom-up tailored nanowires. Nat. Commun. 3, 737 (2012). 22415828 10.1038/ncomms1746 PMC3317500

[b6] Ates S. *et al.* Post-selected indistinguishable photons from the resonance fluorescence of a single quantum dot in a microcavity. Phys. Rev. Lett. 103, 167402 (2009). 19905722 10.1103/PhysRevLett.103.167402

[b7] Juska G., Dimastrodonato V., Mereni L. O., Gocalinska A. & Pelucchi E. Towards quantum-dot arrays of entangled photon emitters. Nat. Photon. 7, 527–531 (2013).

[b8] Munsch M. *et al.* Erratum: dielectric GaAs antenna ensuring an efficient broadband coupling between an InAs quantum dot and a Gaussian optical beam. Phys. Rev. Lett. 111, 239902 (2013). 10.1103/PhysRevLett.110.177402 23679773

[b9] Bulgarini G. *et al.* Nanowire waveguides launching single photons in a Gaussian mode for ideal fiber coupling. Nano Lett. 14, 4102–4106 (2014). 24926884 10.1021/nl501648f

[b10] Borgström M. T., Immink G., Ketelaars B., Algra R. & Bakkers E. P. A. M. Synergetic nanowire growth. Nat. Nanotechnol. 2, 541–544 (2007). 18654364 10.1038/nnano.2007.263

[b11] Dorenbos S. N. *et al.* Position controlled nanowires for infrared single photon emission. Appl. Phys. Lett. 97, 171106 (2010).

[b12] Dalacu D. *et al.* Ultraclean emission from InAsP quantum dots in defect-free wurtzite InP nanowires. Nano Lett. 12, 5919–5923 (2012). 23066839 10.1021/nl303327h

[b13] Hertenberger S. *et al.* Growth kinetics in position-controlled and catalyst-free InAs nanowire arrays on Si (111) grown by selective area molecular beam epitaxy. J. Appl. Phys. 108, 114316 (2010).

[b14] Kang J.-H. *et al.* Defect-free Gaas/AlGaAs core-shell nanowires on Si substrates. Cryst. Growth Des. 11, 3109–3114 (2011).

[b15] Hocevar M. *et al.* Growth and optical properties of axial hybrid III-V/silicon nanowires. Nat. Commun. 3, 1266 (2012). 23232396 10.1038/ncomms2277

[b16] Munshi A. M. *et al.* Position-controlled uniform GaAs nanowires on silicon using nanoimprint lithography. Nano Lett. 14, 960–966 (2014). 24467394 10.1021/nl404376m

[b17] Reimer M. E. *et al.* Electric field induced removal of the biexciton binding energy in a single quantum dot. Nano Lett. 11, 645–650 (2011). 21226507 10.1021/nl1037424

[b18] Minot E. D. *et al.* Single quantum dot nanowire LEDs. Nano Lett. 7, 367–371 (2007). 17298002 10.1021/nl062483w

[b19] Bulgarini G. *et al.* Avalanche amplification of a single exciton in a semiconductor nanowire. Nat. Photon. 6, 455–458 (2012).

[b20] Dousse A. *et al.* Ultrabright source of entangled photon pairs. Nature 466, 217–220 (2010). 20613838 10.1038/nature09148

[b21] Bleuse J. *et al.* Inhibition, enhancement, and control of spontaneous emission in photonic nanowires. Phys. Rev. Lett. 106, 103601 (2011). 21469790 10.1103/PhysRevLett.106.103601

[b22] Akimov I. A., Andrews J. T. & Henneberger F. Stimulated emission from the biexciton in a single self-assembled II-VI quantum dot. Phys. Rev. Lett. 96, 067401 (2006). 16606045 10.1103/PhysRevLett.96.067401

[b23] Singh R. & Bester G. Nanowire quantum dots as an ideal source of entangled photon pairs. Phys. Rev. Lett. 103, 54 (2009). 10.1103/PhysRevLett.103.063601 19792564

[b24] Benson O., Santori C., Pelton M. & Yamamoto Y. Regulated and entangled photons from a single quantum dot. Phys. Rev. Lett. 84, 2513–2516 (2000). 11018923 10.1103/PhysRevLett.84.2513

[b25] Trotta R. *et al.* Highly entangled photons from hybrid piezoelectric-semiconductor quantum dot devices. Nano Lett. 14, 3439–3444 (2014). 24845369 10.1021/nl500968k

[b26] Huber T. *et al.* Polarization entangled photons from quantum dots embedded in nanowires. Preprint at http://arxiv.org/abs/1405.3765 (2014). 10.1021/nl503581d 25395237

[b27] Reimer M. E. *et al.* Overcoming power broadening of the quantum dot emission in a pure wurtzite nanowire. Preprint at http://arxiv.org/abs/1407.2833 (2014).

[b28] Baier M. H, Malko A., Pelucchi E., Oberli D. Y & Kapon E. Quantum-dot exciton dynamics probed by photon-correlation spectroscopy. Phys. Rev. B 73, 205321 (2006).

[b29] Friedler I. *et al.* Solid-state single photon sources: the nanowire antenna. Opt. Expres 17, 2095–2110 (2009). 10.1364/oe.17.002095 19219114

[b30] Young R. J. *et al.* Bell-inequality violation with a triggered photon-pair source. Phys. Rev. Lett. 102, 030406 (2009). 19257332 10.1103/PhysRevLett.102.030406

[b31] Stevenson R. M. *et al.* Evolution of entanglement between distinguishable light states. Phys. Rev. Lett. 101, 170501 (2008). 18999730 10.1103/PhysRevLett.101.170501

[b32] Akopian N. *et al.* Entangled photon pairs from semiconductor quantum dots. Phys. Rev. Lett. 96, 130501 (2006). 16711973 10.1103/PhysRevLett.96.130501

[b33] Young R. J. *et al.* Improved fidelity of triggered entangled photons from single quantum dots. New J. Phys. 8, 29 (2006).

[b34] Hafenbrak R. *et al.* Triggered polarization-entangled photon pairs from a single quantum dot up to 30 K. New J. Phys. 9, 315 (2007).

[b35] Kuroda T. *et al.* Symmetric quantum dots as efficient sources of highly entangled photons: Violation of Bell’s inequality without spectral and temporal filtering. Phys. Rev. B 88, 041306(R) (2013).

[b36] James D. F. V., Kwiat P. G., Munro W. J. & White A. G. Measurement of qubits. Phys. Rev. A 64, 052312 (2001).

[b37] Politi A., Cryan M. J., Rarity J. G., Yu S. & O'Brien J. L. Silica-on-silicon waveguide quantum circuits. Science 320, 646–649 (2008). 18369104 10.1126/science.1155441

[b38] Silverstone J. W. *et al.* On-chip quantum interference between silicon photon-pair sources. Nat. Photon. 8, 104–108 (2014).

[b39] Fedrizzi A., Herbst T., Poppe A., Jennewein T. & Zeilinger A. A wavelength-tunable fiber-coupled source of narrowband entangled photons. Opt. Expres 15, 15377–15386 (2007). 10.1364/oe.15.015377 19550823

[b40] Jayakumar H. *et al.* Deterministic photon pairs and coherent optical control of a single quantum dot. Phys. Rev. Lett. 110, 135505 (2013). 23581338 10.1103/PhysRevLett.110.135505

[b41] Dalacu D. *et al.* Selective-area vapour-liquid-solid growth of InP nanowires. Nanotechnology 20, 395602 (2009). 19724116 10.1088/0957-4484/20/39/395602

[b42] Gulinatti A. *et al.* New silicon SPAD technology for enhanced red-sensitivity, high-resolution timing and system integration. J. Modern Opt. 59, 1489–1499 (2012).

