# Supplementary material for: Observation of strongly entangled photon pairs from a nanowire quantum dot
Source: Nat Commun. 2014 Oct 31;5:5298. doi: 10.1038/ncomms6298 (PMC4220459; doi:10.1038/ncomms6298)
Supplement: Supplementary Information — Supplementary Figures 1-7 and Supplementary Note [file ncomms6298-s1.pdf]

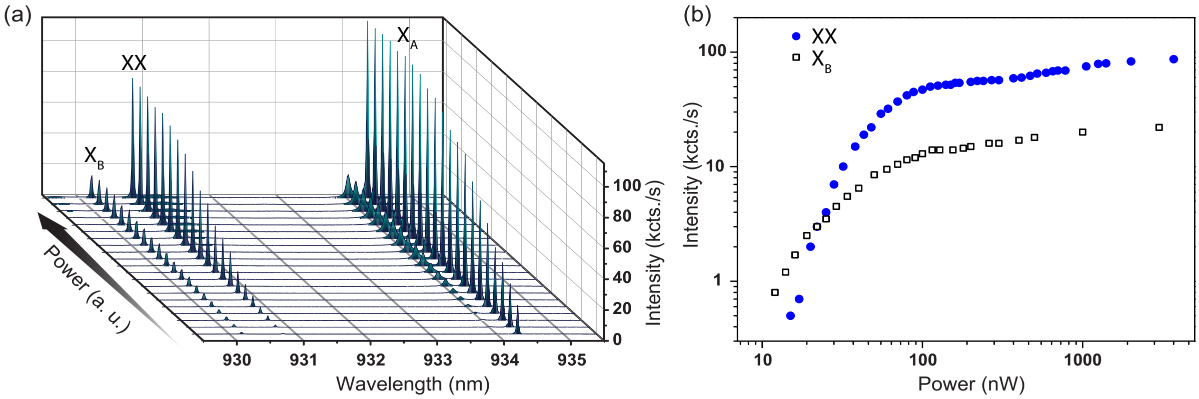

Supplementary Figure 1. Power-dependent measurements of the nanowire quantum dot. (a) Waterfall plot of the quantum transitions for increasing excitation power from 15 nW to 160 nW. The exciton transitions, X_A and X_B, already show luminescence at the lowest power (first row). The biexciton, XX, line starts to appear at higher power and its intensity increases super-linearly. (b) Double logarithmic plot of the single-photon detector counts as a function of excitation power. The XX transition exhibits a steeper slope than the X_B transition. At powers above 140 nW, refilling of the XX state occurs from the quantum dot environment.

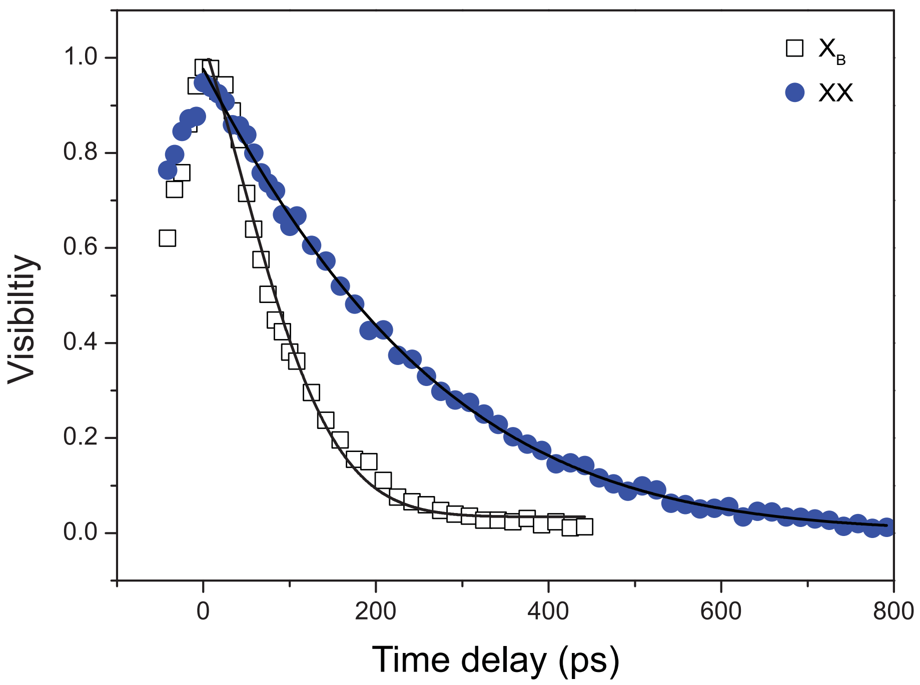

Supplementary Figure 2. Single-photon coherence measurements at 5K for both the biexciton (XX) and exciton (X_B) photon. From a fit to the fringe visibility decay using a Voigt profile (solid lines), described in Supplementary Note 1, we extract an emission linewidth of 1.58 GHz (6.5 μeV) for XX and 4.49 GHz (18.6 μeV) for X_B. Note that the excitation power (100 nW) used for these measurements was near saturation of both the XX and X_B emission.

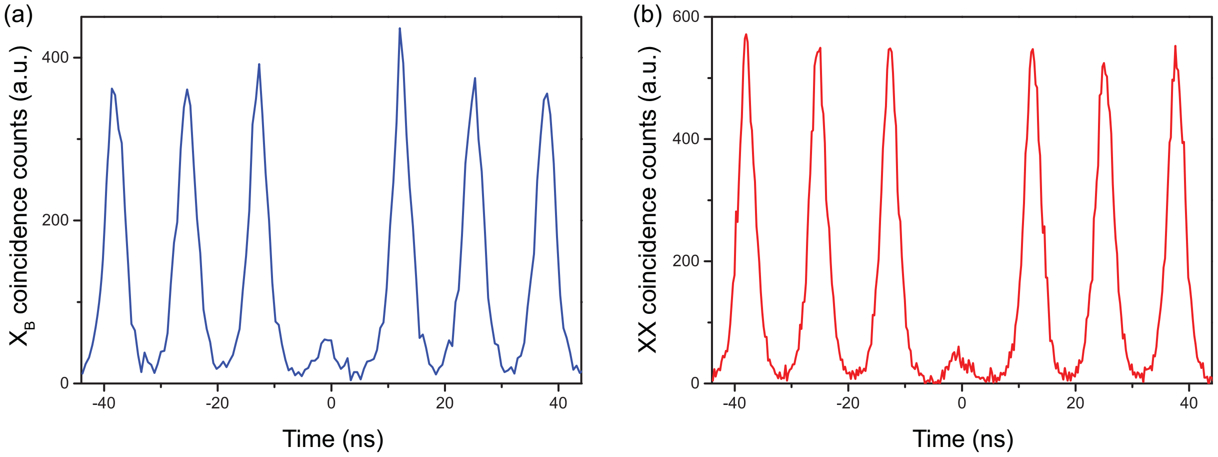

Supplementary Figure 3. Nearly perfect single photon pairs from the biexciton-exciton cascade. Autocorrelation measurements of (a) exciton (X_B) and (b) biexciton (XX) yields a $g^2(0) = 0.07 \pm 0.01$ for X_B and $g^2(0) = 0.04 \pm 0.01$ for XX. These results demonstrate that the nanowire quantum dot generates nearly perfect single photon pairs from the XX-X_B radiative cascade. Note that the excitation power (100 nW) used for these measurements was near saturation of both the XX and X_B emission. The same power was used for the cross-correlation measurements to reconstruct the density matrix.

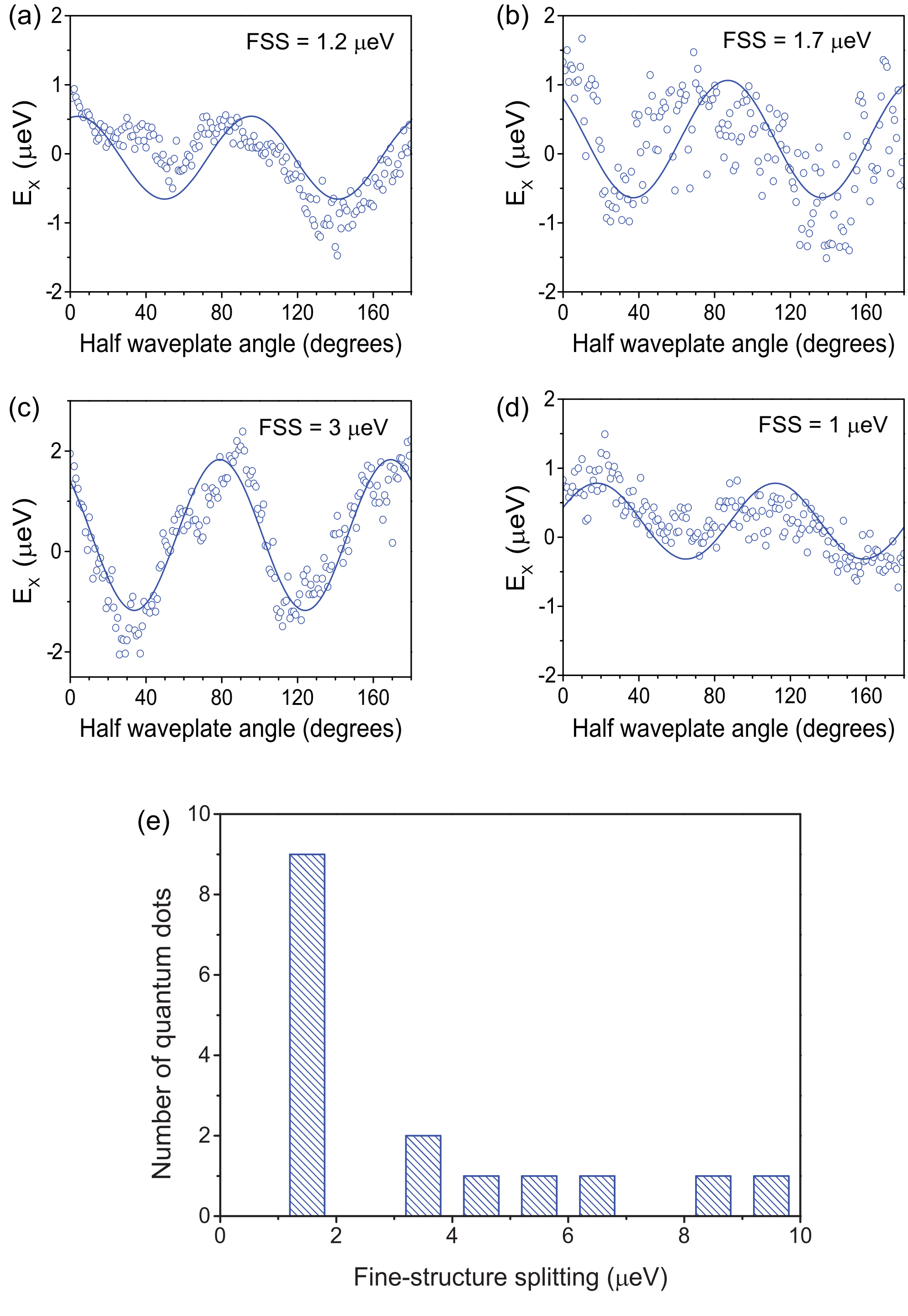

Supplementary Figure 4. Nanowire quantum dots as a low fine-structure splitting system. (a)-(d) Exciton energy, E_X, as a function of half-waveplate polarization angle for four quantum dots. The exciton energy is subtracted for comparison, the mean emission wavelength is ~930 nm. The exciton fine-structure splitting (FSS) is extracted from the sine fit (solid line) to the data (open circles). Note that a sine function fit is needed when the FSS is below the spectral resolution of the experimental setup. (e) Histogram of the FSS for 16 individual quantum dots, exhibiting a mean FSS of 3.4 μeV with a standard deviation of 3.0 μeV. Remarkably, a high percentage (~56%) of the measured quantum dots show a FSS below 2 μeV, thus demonstrating nanowire quantum dots as a low FSS system.

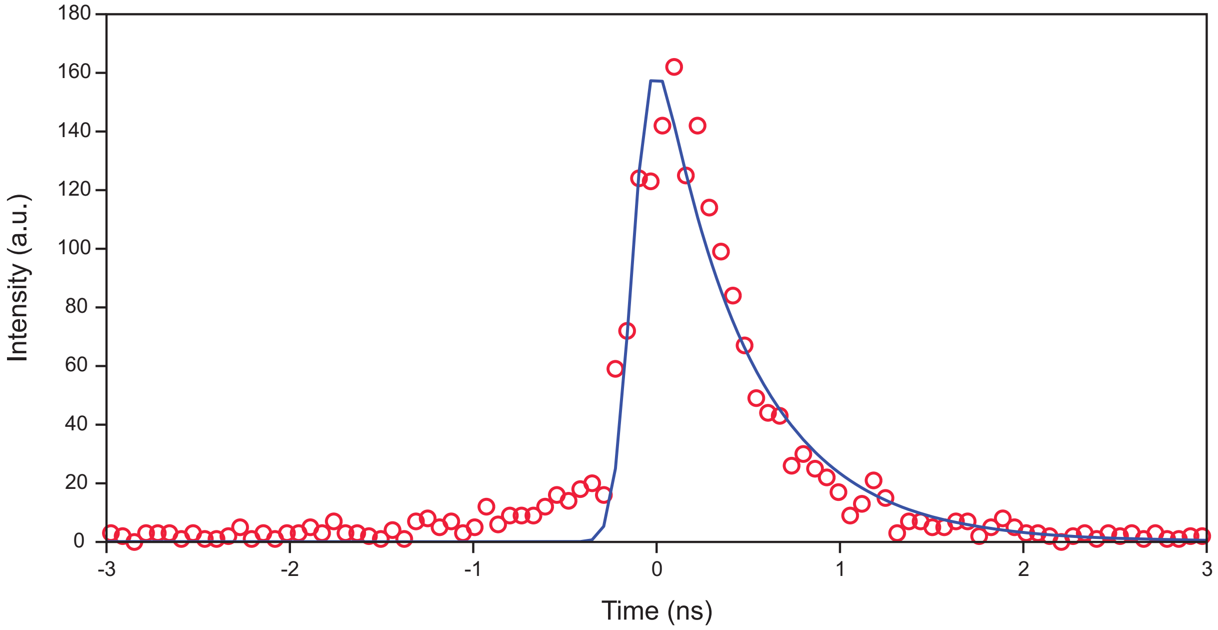

Supplementary Figure 5. Exciton X_B lifetime. Central bunching peak of XX-X_B cross-correlation measurement near zero time delay used to extract the X_B lifetime of investigated nanowire quantum dot. The extracted lifetime is 0.50 ± 0.01 ns from the fit (solid blue line) to the data (open red circles), including the time resolution of the detection system with a full width at half maximum of 80 ps. For this measurement the polarization optics were removed.

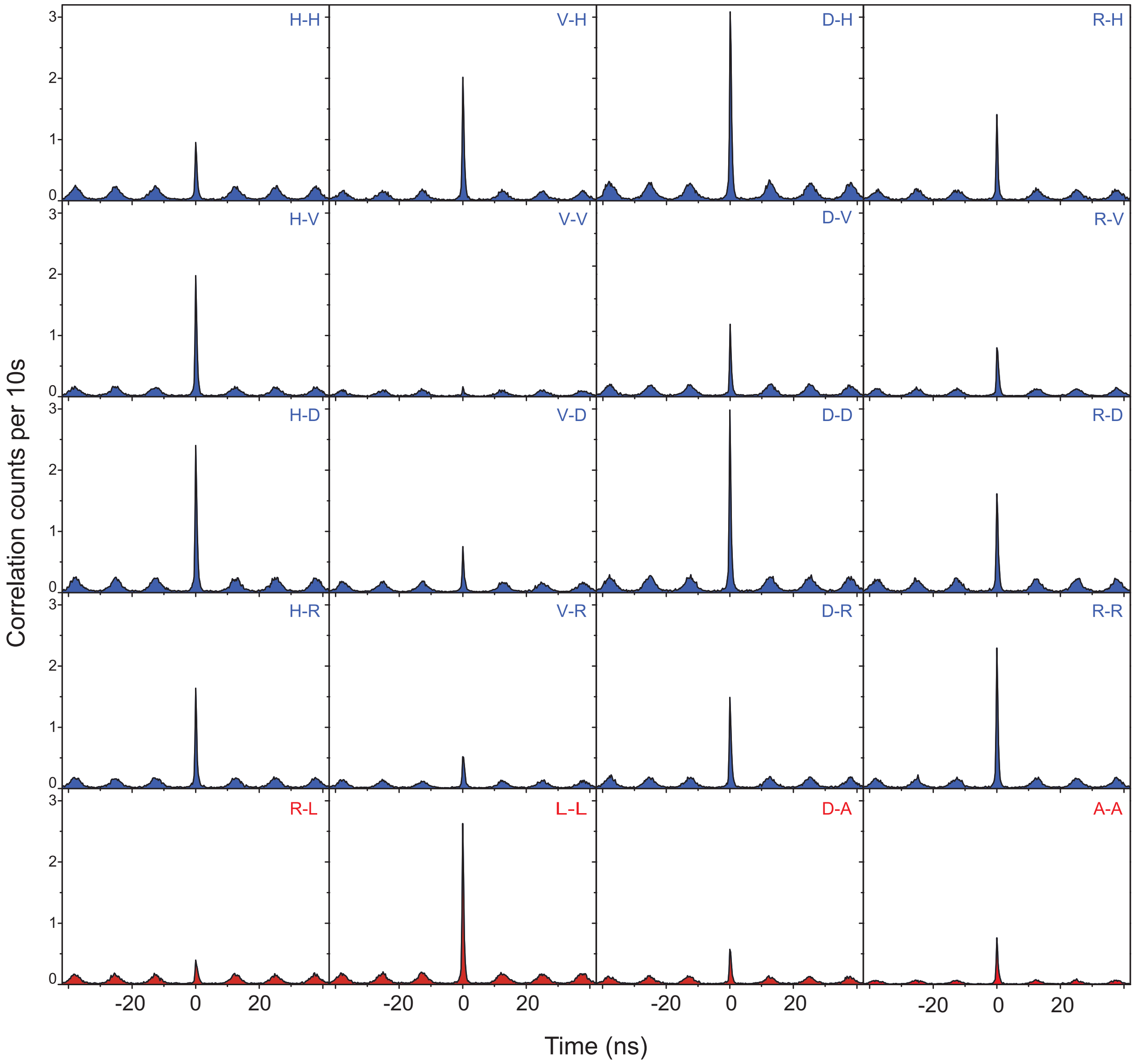

Supplementary Figure 6. Raw cross-correlation measurements of the density matrix. Sixteen cross-correlation measurements (top four rows) of the biexciton and exciton that are needed to reconstruct the density matrix. Start: biexciton; stop: exciton. The first letter stands for the measured polarization of the biexciton photon, whereas the second letter stands for the measured polarization of the exciton photon. We note that the measurements in the bottom row, shown in red, are not used in the density matrix. Additionally, the cross-correlation measurements are not normalized to one, but are depicted in correlation counts per 10s.

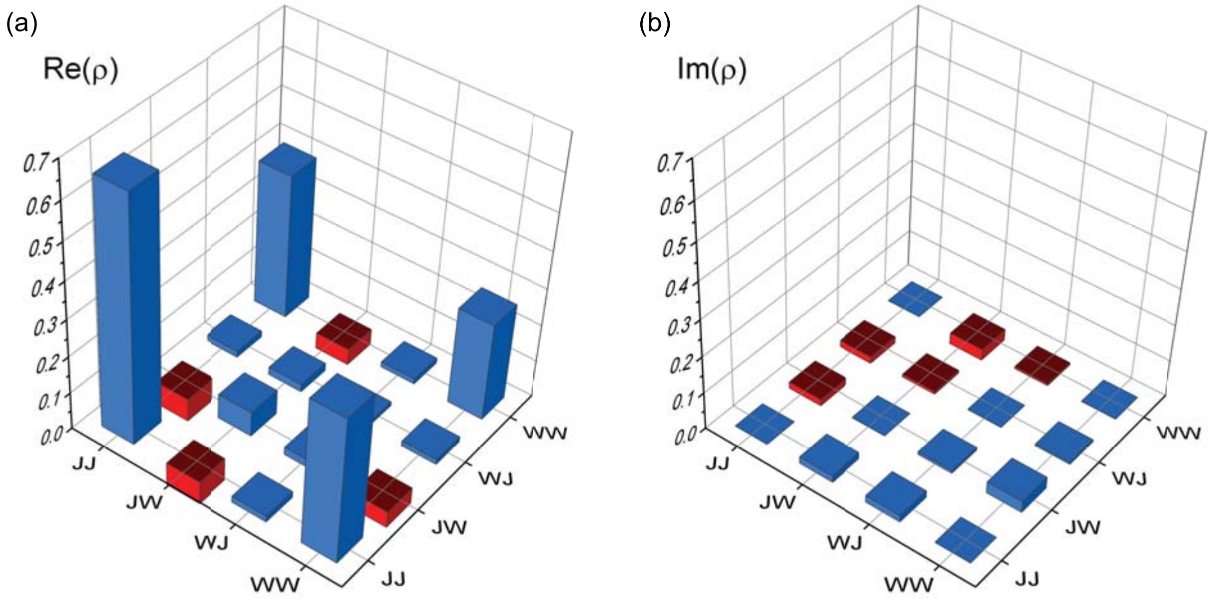

Supplementary Figure 7. Time-gated density matrix in the rotated basis. (a) Real and (b) imaginary part of the density matrix for a time window of 0.13 ns in the rotated basis {*JJ, JW, WJ, WW*}. Here, $J = He^{-i\beta}cos\alpha + Ve^{-i\beta}sin\alpha$ and $W = -He^{i\beta}sin\alpha + Ve^{i\beta}cos\alpha$ are orthogonal elliptical polarizations, with $\alpha = 36^0$ and $\beta = 50^0$. The time-gated entangled photons yield a fidelity of 0.854 ± 0.006 to the two-photon state $(|JJ\rangle + |WW\rangle)/\sqrt{2}$ and a concurrence of 0.80 ± 0.02. The positive matrix elements are blue and the negative matrix elements are red.

Supplementary Note 1. Single-photon coherence for exciton and biexciton

The quantum dot emission linewidths were investigated by field-correlation measurements using a Michelson interferometer. This technique not only provides information about the emission linewidth, but also reveals the lineshape owing to charge fluctuations in the quantum dot environment. The spectral lines have a Voigt profile, which is a Gaussian convolved with a Lorentzian. By fitting the decay of the fringe visibility in Supplementary Fig. 2 with a Voigt profile in the time domain, which is a product of a Gaussian and Lorentzian,

$$g^1(\tau) \sim \exp\left[-\frac{\pi}{2}\left(\frac{\tau}{\tau_c}\right)^2 - \frac{|\tau|}{T_2}\right],$$

we extract a homogeneous coherence time, T_2, of 281 ± 81 ps for XX and 200 ± 25 ps for X_B, whereas the coherence time for the Gaussian component, τ_c, is 813 ± 76 ps for XX and 187 ± 13 ps for X_B. In the frequency domain the full-width at half-maximum of the resulting Voigt profile is

$$\Delta f_v = 0.535\Delta f_L + \sqrt{0.217\Delta {f_L}^2 + \Delta {f_G}^2},$$

where $\Delta f_L = 1/\pi T_2$ is the full-width at half-maximum of the Lorenztian lineshape, and $\Delta f_G = \sqrt{2ln2}/\sqrt{\pi}\tau_c$ is the full-width at half-maximum of the Gaussian lineshape, with τ_c being the coherence time of the Gaussian component. Thus, including Gaussian line broadening the emission linewidth is 1.58 GHz (6.5 μeV) for XX and 4.49 GHz (18.6 μeV) for X_B.

The observed Gaussian line broadening is due to charge fluctuations in the quantum dot environment that lead to spectral wandering. This process occurs during the time needed to acquire each visibility data point in the field correlations measurements presented in Supplementary Fig. 2 (~10 minutes in our measurement).
